# Supernumerary, ectopic tooth in the maxillary antrum presenting with recurrent haemoptysis

**DOI:** 10.1186/1746-160X-6-26

**Published:** 2010-11-11

**Authors:** Taimur Saleem, Umair Khalid, Anam Hameed, Shehzad Ghaffar

**Affiliations:** 1 Medical College, Aga Khan University, Stadium Road, Karachi 74800, Pakistan; 2 Section of Otolaryngology/Head and Neck Surgery, Department of Surgery, Aga Khan University, Stadium Road, Karachi 74800, Pakistan

## Abstract

**Background:**

Ectopic eruption of teeth in non-dental sites is a rare phenomenon and can present in a variety of ways such as chronic or recurrent sinusitis, sepsis, nasolacrimal duct obstruction, headaches, ostiomeatal complex disease and facial numbness. However, presentation of such patients with recurrent haemoptysis has not been described in the literature so far. We have described a case of an ectopic, supernumerary molar tooth in the maxillary antrum in a patient who initially presented with haemoptysis.

**Case presentation:**

A 45-year-old male presented with a 2-month history of episodic haemoptysis. A pedunculated growth from the inferior nasal turbinate was seen with fibre-optic visualization. Although the patient was empirically started on antibiotic and anti-allergic therapy, there was no improvement after a few weeks and the patient had recurrent episodes of haemoptysis. Fibre-optic visualization was repeated showing bilateral osteomeatal erythema. Computed tomography scan of the paranasal sinuses demonstrated complete opacification of the left maxillary antrum along with a focal area of density comparable to bone. An ectopic, supernumerary molar tooth was found in the left maxillary antrum on endoscopic examination and subsequently removed. In addition, copious purulent discharge was seen. Post-operatively, the patient was treated with a 10-day course of oral amoxicillin-clavulanate. On follow-up, he reported resolution of symptoms.

**Conclusion:**

Recurrent haemoptysis has not been described as a presentation for a supernumerary, ectopic tooth in literature before. We recommend that in patients with sinusitis-type of opacification of maxillary antrum and whose condition is refractory to conventional medical treatment, consideration should be given to the investigation of possible underlying anomalies as the cause of such symptoms. Presence of foreign bodies and ectopic teeth in paranasal sinuses can be reliably excluded with the use of appropriate radiological imaging and endoscopic examination.

## Background

Ectopic and supernumerary teeth have been rarely described in non-dental and non-oral sites such as the mandibular condyle, coronoid process, orbit, palate, nasal cavity, nasal septum, chin and the maxillary antrum [1,2]. In contrast, dental impressions or affections may be commonly seen within the maxillary sinus [3].

We hereby describe a case of a 45-year-old man with a supernumerary, ectopic molar tooth in the left maxillary antrum who initially presented with haemoptysis. To the best of our knowledge, this is the first reported case of such a presentation.

## Case history

A 45-year-old male initially presented to our institution with a 2-month history of episodic haemoptysis. The blood was scanty in amount and comprised largely of clots. The patient denied any history of cough, nasal irritation, hoarseness, febrile illness or weight loss. He had never smoked tobacco in his life; however, he occasionally chewed *paan *(betel quid without tobacco). His past surgical history was significant for surgical correction of deviated nasal septum 20 years ago and excision of a benign vocal cord nodule 6 years ago. He did not recall any history of spontaneous or iatrogenic trauma in the ear, nose and throat regions. His wife had a history of treated pulmonary tuberculosis.

The patient was vitally stable; general and systemic examinations were also unremarkable. Apart from a small, pedunculated growth from the inferior nasal turbinate, the fibre-optic visualization did not reveal any aberrancy or abnormalities. He was tentatively started on outpatient antibiotic and anti-allergic therapy.

**Figure 1:**
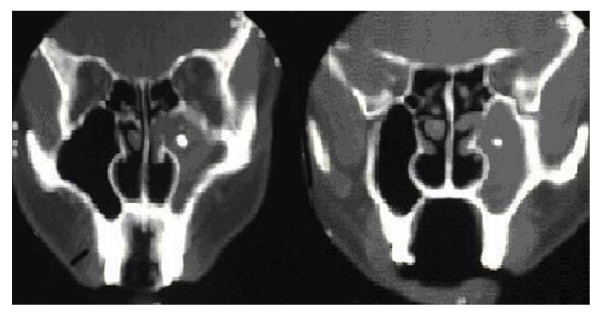
**Computed tomography scan images showing complete opacification of left maxillary antrum along with widening of the ostium**. A small, round high density body is also seen in the centre of the antrum.

However, this treatment regimen did not alleviate the symptoms as he returned after a few months with recurrent haemoptysis. Physical examination did not reveal any new finding. Fibre-optic visualization at this instance showed evidence of bilateral osteomeatal erythema. Computed tomography (CT) scan of the paranasal sinuses demonstrated complete opacification of the left maxillary antrum along with marked widening of its ostium. Mucosal thickening of the left ethmoidal cells was also noted. There was a focal area within the left maxillary sinus which displayed a density comparable to that of bone. (**Figure **1).

Based on these findings, the patient was scheduled for the endoscopic removal of a potential foreign body along with debridement of the left maxillary sinus. Limited pre-operative laboratory testing showed no abnormalities. Uncinectomy was done and anterior ethmoid cells were opened up. Intra-operatively, an ectopic molar tooth was found in the left maxillary antrum and subsequently removed; the patient had a full set of 32 permanent teeth in his buccal cavity. In addition, copious purulent discharge was seen which was sent for culture and sensitivity studies. Polypoidal mucosa was also resected and sent for histopathology.

The sample of the purulent discharge grew penicillin sensitive *Streptococcus milleri*. The histopathology report of the polypoid mass showed benign features. Post-operatively, the patient was treated with a 10-day course of oral amoxicillin-clavulanate. The patient visited the outpatient clinic once for follow-up. At that time, he was well and had no active complaints.

## Discussion

Ectopic tooth in the maxillary sinus is a rare phenomenon. A recent review by Lamb et al identified only 35 reported cases of this phenomenon in English language medical literature since 1927 [4].

### 1. Pathogenesis of ectopic, supernumerary teeth

The process of tooth development is the corollary of complex interactions between the oral epithelium and the underlying mesenchymal tissue. If abnormal tissue interactions disrupt the process, the result is ectopic tooth development and eruption [2]. However, the etiology of ectopic teeth in maxillary antrum is not yet entirely clear. Some reports have highlighted the role of benign odontogenic cysts called dentigerous cysts in the appearance of ectopic teeth. These cysts are epithelial-lined developmental cavities at the cementoenamel junction that arise from the enamel organ after amelogenesis is complete. They are thus associated with the crowns of permanent teeth and may displace the teeth into ectopic positions such as the maxillary sinus [5,6]. We examined the surrounding soft tissue thoroughly but did not find any evidence of such a cyst in the proximity of the ectopic tooth in our patient. Also, the tooth in our patient was supernumerary which makes the association rarer, although not impossible [7]. In literature, dentigerous cysts are mostly associated with unerupted teeth and come to notice during the investigation of failure of tooth eruption, a missing tooth or misaligned tooth [6]. Other etiologies of ectopic teeth in maxillary sinus include trauma/iatrogenic activity, developmental anomalies and idiopathic etiology [2,4]. Crowding of teeth in the buccal cavity has also been described as an etiologic factor for appearance of ectopic teeth in maxillary sinus [8]. In our patient, the etiology of the ectopic tooth was most probably idiopathic.

### 2. Clinical presentation

Ectopic teeth in paranasal sinuses can present with a variety of clinical manifestations. Literature review showed that patients with such teeth may present with recurrent or chronic sinusitis [2,9-11], sepsis, nasolacrimal duct obstruction, ostiomeatal complex obstruction [4], headaches and facial numbness [3]. Our patient presented with recurrent haemoptysis; however, this presentation has not been described in literature so far.

### 3. Diagnosis and management

Sinus disease associated with ectopic teeth may be refractory to conventional treatment such as simple antibiotic or antihistamine therapy [2,4,9]. We initially offered the patient conventional treatment for allergic rhinosinusitis. A thorough intraoral and otorhinolaryngologic examination did not reveal any significant abnormalities. A CT scan was performed when the patient's symptoms persisted despite empirical medical treatment. The diagnosis of a foreign body with radio-density similar to bone was made on the basis of CT scan. In literature, CT scan study is indicated when the ectopic tooth is associated with an antral mass and prior to surgery [4]. In one of the largest series of patients with ectopic teeth in maxillary sinus (n = 14), Water's view (also known as semi-axial or occipitomental view) of plain-film radiography was found to be an inadequate imaging study in the diagnosis of ectopic teeth [8]. In contrast, CT scan (axial and coronal section) has been found to provide excellent features of teeth in maxillary sinus in one series of 12 patients from Israel [12].

Complete opacification of the left maxillary antrum was seen in our patient. In literature, the presence of maxillary sinus fluid has been correlated with a higher incidence of odontogenic etiology [9,13]. We performed endoscopic enucleation of the ectopic tooth and debridement of the maxillary antrum. Literature has described surgical management of such teeth via either Caldwell-Luc operation or endoscopic approach [2,4,14,15]. The endoscopic approach is associated with lesser operative and post-operative morbidity [15]. Our patient was also discharged early without any post-operative or intra-operative complications.

## Conclusion

In summary, we have described a patient with an ectopic, supernumerary tooth in the maxillary sinus who presented with recurrent haemoptysis. Overt sinusitis, however, was not clinically present. Such a presentation has not been described in literature before. We recommend that in patients with sinusitis-type opacification of maxillary antrum and whose condition is refractory to conventional medical treatment, consideration should be given to the investigation of possible underlying anomalies as the cause of such symptoms. Presence of foreign bodies and ectopic teeth in paranasal sinuses can be reliably excluded with the use of appropriate radiological imaging and endoscopic examination.

## Consent

Written, informed consent was obtained from the patient for the publication of this case report and accompanying images. A copy of the consent form is available for review by the Editor-in-Chief of this journal.

## Competing interests

The authors declare that they have no competing interests

## Authors' contributions

TS, UK and AH were involved in data collection, interpretation and writing the manuscript. SG was involved in study conception and design, drafting the manuscript and providing overall supervision in the project. All authors read and approved the final manuscript.
